# Phylogeny affects host's weight, immune response and parasitism in damselflies and dragonflies

**DOI:** 10.1098/rsos.160421

**Published:** 2016-11-09

**Authors:** Jaakko J. Ilvonen, Jukka Suhonen

**Affiliations:** Section of Ecology, Department of Biology, University of Turku, 20014 Turku, Finland

**Keywords:** host–parasite associations, immune defence, parasitism, insect, encapsulation

## Abstract

Host–parasite interactions are an intriguing part of ecology, and understanding how hosts are able to withstand parasitic attacks, e.g. by allocating resources to immune defence, is important. Damselflies and dragonflies show a variety of parasitism patterns, but large-scale comparative immune defence studies are rare, and it is difficult to say what the interplay is between their immune defence and parasitism. The aim of this study was to find whether there are differences in immune response between different damselfly and dragonfly species and whether these could explain their levels of gregarine and water mite parasitism. Using an artificial pathogen, a piece of nylon filament, we measured the encapsulation response of 22 different damselfly and dragonfly species and found that (i) there are significant encapsulation differences between species, (ii) body mass has a strong association with encapsulation and parasite prevalences, (iii) body mass shows a strong phylogenetic signal, whereas encapsulation response and gregarine and water mite prevalences show weak signals, and (iv) associations between the traits are affected by phylogeny. We do not know what the relationship is between these four traits, but it seems clear that phylogeny plays a role in determining parasitism levels of damselflies and dragonflies.

## 1. Introduction

The host–parasite interaction is an interaction between the host's defence and parasite's offensive capabilities. The host has a variety of defensive measures, the immune system being one of the most important. An effective immune system is a complex mechanism, with specific and non-specific responses targeted and activated in the presence of foreign objects. The invertebrate innate immune system consists of, for example, phagocytosis, anti-microbial peptides and melanization [1]. In addition, a few studies have shown that invertebrates may have a certain immune mechanism that could be classified as an ‘induced’ immunity, which may be more protective upon secondary pathogen exposure [2,3]. The melanin-producing enzyme cascade (phenoloxidase (PO)) and regulation of these PO levels in invertebrates are responsible for wound healing and encapsulating foreign objects [1,4,5] such as the feeding tubes of larval water mites (Arrenuridae) [6,7]. These defence mechanisms add up to an effective immune system, capable of defending against parasites and thus prolonging the host's lifespan and reproductive success.

We know from previous studies that the strength of immune response may vary within and between species [8–12], but contradictory results have also been found [13]. There are studies [2,11] that have found immunological responses targeted specifically against a certain type of parasitic species. Host species with the highest water mite prevalence and intensity have been found to have the highest probability of mounting an encapsulation [6,14]. It has also been found that individuals with a higher degree of genetic diversity can protect themselves more efficiently against parasites than hosts with less genetic diversity [15–17]. In addition, individuals may choose to allocate resources from immune function to other essential mechanisms. During reproduction, males and females may be able to divert resources from immunity for use in reproductive processes and vice versa [18], or they can divert resources to immunity when in the presence of predators [19]. Malnourishment has been found to reduce the body's fat reserves which downregulates immune functions, thus reducing the production of anti-microbial peptides [3,20] or the host's ability to melanize [21]. Moreover, a study of damselflies found that encapsulation was stronger later in the flight season compared with at an earlier time [7].

However, these studies have had a specific viewpoint on immune response without the need to look for a broader and larger pattern. Recently, it was discovered [22] that parasitism levels vary greatly between closely related species, and it was suggested that differences in immune response were the most probable reason for this difference. Unfortunately, because large comparative immune response studies using closely related species do not exist, forming a general hypothesis of immune response and its relationship with parasitism has not been possible.

In this study, we tried to find whether immune response levels within the Odonata order followed a pattern that could be generalized into explaining parasite infections in odonates. First, we determined the average fresh body mass, strength of immune response and ecto- and endoparasite levels of different damselfly (Zygoptera) and dragonfly (Anisoptera) species. Then we evaluated how these factors may have affected each other and whether phylogeny might explain possible differences between species. An odonate species was used as an independent sample and individuals' ability to encapsulate a foreign (non-self) object was used to quantify the level of immune response for each species. To measure the immune response, we used an artificial nylon implant, because at the time this was one of the best methods of reflecting a natural immune response and variation of water mite encapsulation [23,24] (but see also [1,14]). To assess parasitism, we measured the average prevalence of endo- and ectoparasites for each odonate species. We also controlled for the phylogenetic relatedness of different odonate species in the measured traits. Based on previous findings [14,22], we predicted considerable differences in the encapsulation rates between different odonate species. We also predicted that because of the observed differences in parasitism between damselflies and dragonflies [22], immune response levels were likely to follow this pattern and phylogeny might explain these differences.

## 2. Material and methods

Common and relatively widespread damselfly (Zygoptera) and dragonfly (Anisoptera) species (forming the order Odonata) were chosen for this study, because they have been studied extensively in the past, their ecology is well understood, they have a wide range of measurable attributes (e.g. body mass, behaviour and immune response) and they are widely parasitized insects harbouring both endoparasitic gregarines (Apicomplexa: Eugregarinorida) and ectoparasitic water mites (Acari: Hydrachnida) [22,25].

### 2.1. Fieldwork

Field data were collected mainly from Central and Southern Finland from 2009 to 2013 consisting of 12 damselfly and 10 dragonfly species (table 1). Specimens for each species were collected within a few days from the same location to minimize spatial and temporal variation. Our aim was to collect an equal number of adult males and females for each damselfly and dragonfly species to reduce the difference between sexes in the measured traits. Adults were separated from juveniles by the stiffness of their wings [26]. Individuals were collected between 10 : 00 and 16 : 00 using a sweeping net, sexed, placed in an individual container with a moist paper towel to avoid dehydration and transported to a laboratory for further tests.

**Table 1.** Estimated marginal means of fresh body mass, encapsulation response and gregarine and water mite prevalences of different damselfly (*Z*) and dragonfly (*A*) species derived from the GLMs. Term mg stands for milligrams, s.e. indicates standard error of mean, *n* represents the number of individuals evaluated, ER represents the amount of encapsulation of the implant and % indicates prevalence. Order of species is representative of their phylogeny used in this study.

	weight			encapsulation			gregarines			water mites		
suborder/species	mg	s.e.	*n*	ER	s.e.	*n*	%	s.e.	*n*	%	s.e.	*n*
A/*Sympetrum vulgatum*	221.2	9.3	18	23.5	2.6	18	0	0.0	18	0	0	18
A/*Sympetrum flaveolum*	157.9	5.3	27	26.7	2.4	27	8	5.2	21	4	3.7	21
A/*Sympetrum danae*	140.5	3.9	39	23.8	1.8	39	0	0.0	41	0	0	41
A/*Leucorrhinia dubia*	149.9	5.1	28	30.0	2.7	28	0	0.0	28	3	3.2	28
A/*Libellula quadrimaculata*	426.3	28.9	7	21.9	3.9	7	0	0.0	7	0	0	7
A/*Somatochlora metallica*	411.4	20.6	13	23.7	3.1	13	0	0.0	13	0	0	13
A/*Cordulia aenea*	363.1	14.9	18	21.5	2.4	18	0	0.0	19	0	0	19
A/*Aeshna grandis*	873.1	29.5	25	23.5	2.2	25	0	0.0	28	0	0	28
A/*Aeshna juncae*	780.5	27.9	24	23.7	1.9	35	0	0.0	36	0	0	36
A/*Aeshna subarctica*	712.5	27.2	19	24.2	2.1	28	0	0.0	31	0	0	31
Z/*Ischnura elegans*	40.6	1.5	19	10.4	1.2	16	0	0.0	22	7	5.2	22
Z/*Enallagma cyathigerum*	35.4	1.3	22	21.9	2.2	22	24	8.7	24	17	7.7	24
Z/*Erythromma najas*	61.8	2.0	31	18.9	1.6	31	92	4.6	31	98	1.9	31
Z/*Coenagrion hastulatum*	33.9	1.3	22	17.0	1.7	22	63	10.4	22	100	0	23
Z/*Coenagrion pulchellum*	36.1	1.3	22	15.8	1.6	22	12	6.5	24	34	10.2	24
Z/*Coenagrion armatum*	32.3	1.1	20	21.3	2.2	20	20	7.4	29	88	5.9	29
Z/*Coenagrion johanssoni*	20.3	0.7	27	11.2	1.0	26	57	9.3	29	21	8	29
Z/*Calopteryx virgo*	175.5	7.0	19	15.2	1.6	19	91	6.4	20	0	0	20
Z/*Calopteryx splendens*	134.0	5.1	22	20.1	2.0	22	0	0.0	22	0	0	22
Z/*Platycnemis pennipes*	48.0	1.7	26	21.5	2.0	26	56	9.9	26	4	3.5	26
Z/*Pyrrhosoma nymphula*	55.9	2.0	24	22.1	2.1	24	100	0.0	24	10	6	24
Z/*Lestes sponsa*	48.8	1.8	23	20.8	2.0	23	4	4.1	23	0	0	23
number of individuals			495			512			538			539

### 2.2. Laboratory work

In the laboratory, the effectiveness of the immune system, i.e. encapsulation rate, was tested for 512 odonate individuals by inserting a 3 mm long nylon filament (sterilized with 99.5% ethanol; henceforth called implant) into the second abdominal pleura on the dorsal side of the sternal–tergal margin [27]. This treatment stimulated the encapsulation response, mimicking the penetration of a parasite's feeding tube [23,24]. Temperature in the laboratory was kept constant, in order to avoid affecting individuals' ability to mount an immunological response. Owing to melanization, cell layers were formed on the nylon filament, thus making it darker. The darker the nylon filament became, the stronger the immune response. Implanted specimens were held in individual containers for 18 h in order for the individuals to encapsulate the implant thoroughly and to empty their digestive tracts. This was done to minimize the possible weight differences caused by ingestion of prey prior to capture. After 18 h, implants were removed and the fresh body mass of 495 odonate individuals was measured using a Mettler AT-20 analytical balance to the nearest milligram. Afterwards, both sides of the implants were photographed and analysed using ImageJ v. 1.47 software to determine the individual level of immune response by measuring the mean grey value of the implant. Using ImageJ, we selected two areas from the two sides of the implant: ‘C’ as the control end of the implant located on the outside of the specimen and ‘S’ as the part of the implant that was inside the specimen. While a particular area was selected, we used the Measure function to determine the Mean Grey value of that particular area. Using the following formula:

2.1 $$IR=\frac{\left(CL-SL)+(CR-SR\right)}{2},$$

where ‘*C*’ is the control end of the implant located on the outside of the specimen, ‘*S*’ is the end that has been inside the specimen, i.e. the encapsulated part, ‘*L*’ is the left side of the implant and ‘*R*’ is the right side of the implant, we were able to get a value indicating individuals' strength of immune response. If the control end of the implant was also melanized, we calculated the average control value of that particular species and sex and used it as the control value.

The difference in sample sizes between encapsulation rate and weight was due to the inability to measure these proxies from certain individuals.

After the removal of the implant, attached larval water mites were counted using a dissecting microscope. This was followed by decapitation of the specimens, dissection of their digestive tracts and the enumeration of gregarines using a microscope.

### 2.3. Statistics

We used generalized linear models (GLMs) to test whether significant differences existed between species and sexes in fresh body mass, encapsulation response and both parasite prevalences. In the GLMs of fresh body mass and encapsulation, the link functions were gamma with log links, because the data were highly skewed. In the gregarine and water mite GLMs, the link functions were log links and the distributions were binomial (1 = parasitized individual, 0 = non-parasitized individual). All the following statistical tests for each damselfly and dragonfly species were based on the estimated marginal means derived from these GLMs.

Because the fresh body mass data of each species did not fit the assumptions of parametric tests and to improve the readability of the figures, the data were transformed to log_10._ To see whether the suborder differences in fresh body mass, encapsulation response and gregarine and water mite parasitism were significant we used a Mann–Whitney *U*-test. This test was used because the variances between the two suborders differed from each other and *t*-test could not be used. Regression analysis was used to see whether relationships between the different measured attributes were significant between species. Statistical tests were analysed with IBM SPSS Statistics v. 22.

For phylogenetic analysis, we used R Studio (v. 0.99.903) and the following packages: ‘picante’, ‘ape’, ‘adephylo’, ‘ade4’, ‘phylobase’, ‘geiger’, ‘phytools’ and ‘caper’. First, we created a phylogenetic tree of the 22 different odonate species using Carle *et al*. [28] for the Anisopteran species, except for the three *Sympetrum* species which were organized according to Pilgrim & von Dohlen [29], and using Dumont *et al*. [30] for the Zygopteran species, except for the four coenagrionids which were organized according to O'Grady & May [31]. Branch lengths were standardized to one, because actual lengths were unknown. The tree was drafted using Boc *et al*. [32] and can be seen in the electronic supplementary material, appendix S1. Then we used Pagel's *λ* [33] as a measure to calculate whether a phylogenetic signal exists in weight, encapsulation or either type of parasitism. This was done in order to find whether the differences between species in the traits measured follow a Brownian motion model of evolution, i.e. whether phylogeny alone can explain these differences. Phylogenetic generalized least-square models (PGLS) were used to compare fresh body mass, encapsulation response and both types of parasitism in the light of their species' close relatedness. Formulae used in R Studio can be seen in the electronic supplementary material, appendix S2.

## 3. Results

As expected, there were huge differences in the fresh body mass between different odonate species (table 1). The lightest species was the damselfly *Coenagrion johanssoni* (20.3 ± 0.7 mg, mean ± s.e.) and the heaviest was the dragonfly *Aeshna grandis* (873.1 ± 29.5 mg; table 1). Damselflies were significantly lighter (60.2 ± 13.9 mg, *n* = 12) than dragonflies (423.6 ± 87.1 mg, *n* = 10; Mann–Whitney *U*-test: *U* = 3, *p* < 0.001). Mass was significantly different between species (GLMs, Wald = 20850.0, d.f. = 21, *p* < 0.001) and sexes (GLMs, Wald = 276.7, d.f. = 1, *p* < 0.001), females being heavier than males. Fresh body mass showed a very strong and significant phylogenetic signal (Pagel's *λ*, *λ* = 1.0, *p* < 0.001).

**Figure 1.**
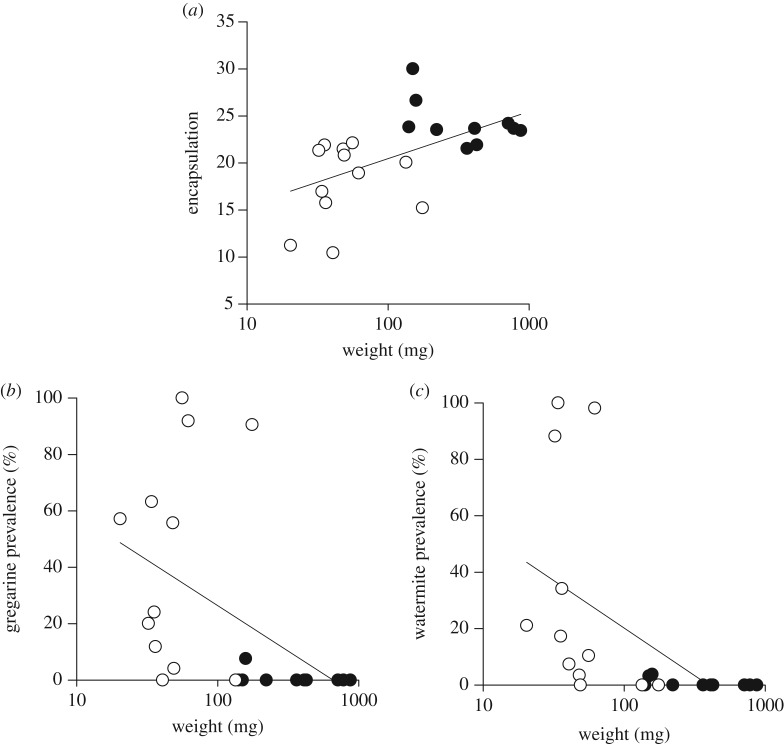
Encapsulation response (*a*), gregarine prevalence (*b*) and water mite prevalence (*c*) in relation to the average fresh body mass (weight) of different odonate species without phylogenetic corrections. White dots represent damselflies (Zygoptera) and black dots represent dragonflies (Anisoptera).

We found considerable differences in the encapsulation response between different odonate species (table 1). Damselfly *Ischnura elegans* had the lowest encapsulation rate, whereas dragonfly *Leucorrhinia dubia* had the highest (table 1). At the suborder level, damselflies had a lower encapsulation rate (18.0 ± 1.2, *n* = 12) than dragonflies (24.3 ± 0.8, *n* = 10, Mann–Whitney *U*-test: *U* = 3, *p* < 0.001; figure 1*a*). Encapsulation response was different between species (GLMs, Wald = 139.3, d.f. = 21, *p* < 0.001; figure 1*a*), but not between sexes (GLMs, Wald = 0.0, d.f. = 1, *p* = 0.998). Encapsulation response had a weak but significant phylogenetic signal (Pagel's *λ*, *λ* = 0.44, *p* = 0.008).

Similar to our previous findings [22], both gregarine and water mite parasitism showed considerable differences between different odonate species and between the two suborders (table 1). Gregarine prevalence ranged from 0 to 100%, as did water mite prevalence (table 1). At the suborder level, damselflies had a significantly higher gregarine prevalence (43.3 ± 10.9%, *n* = 12) compared with dragonflies (0.8 ± 0.8%, *n* = 10, Mann–Whitney *U*-test: *U* = 99, *p* = 0.009; figure 1*b*). Damselflies had also a much higher water mite prevalence (31.6 ± 11.5%, *n* = 12) compared with dragonflies (0.7 ± 0.5%, *n* = 10, Mann–Whitney *U*-test: *U* = 101, *p* = 0.006; figure 1*c*). Gregarine prevalence was different between species (GLMs, Wald = 68.4, d.f. = 21, *p* < 0.001), but not between sexes (GLMs, Wald = 3.3, d.f. = 1, *p* = 0.71), even though males were less often infected. Water mite prevalence was different between both species (GLMs, Wald = 67.8, d.f. = 21, *p* < 0.001) and sexes (GLMs, Wald = 11.3, d.f. = 1, *p* = 0.001), males being less often infected with water mites. Both gregarine prevalence (Pagel's *λ*, *λ* = 0.44, *p* = 0.044) and water mite prevalence (Pagel's *λ*, *λ* = 0.48, *p* = 0.039) showed weak but significant phylogenetic signals.

We also tested whether significant associations existed between the different attributes measured with and without taking phylogeny into account. Without taking phylogeny into account fresh body mass had a strong association with encapsulation response (regression analysis, *F*_1,20_ = 8.68, *p* = 0.008), gregarine prevalence (*F*_1,20_ = 5.68, *p* = 0.027) and water mite prevalence (*F*_1,20_ = 7.5, *p* = 0.013). However, encapsulation response did not show a significant correlation between gregarine prevalence (*F*_1,20_ = 3.437, *p* = 0.079) or water mite prevalence (*F*_1,20_ = 1.74, *p* = 0.202). Interestingly, gregarine prevalence had a significant association with water mite prevalence (*F*_1,20_ = 5.42, *p* = 0.03).

When phylogeny was taken into account, the relationships between fresh body mass and encapsulation (PGLS, *F*_1,20_ = 0.118, *p* = 0.734), gregarine prevalence (*F*_1,20_ = 1.261, *p* = 0.275) or water mite prevalence (*F*_1,20_ = 0.168, *p* = 0.686) lost their significance. Relationships between encapsulation response and gregarine prevalence (*F*_1,20_ = 0.108, *p* = 0.745) or water mite prevalence (*F*_1,20_ = 1.458, *p* = 0.241) were also not significant. The relationship between gregarine and water mite prevalences was also not significant (*F*_1,20_ = 0.034, *p* = 0.856).

## 4. Discussion

In this study, we found four main results. First, considerable variations in the encapsulation response between different dragonfly and damselfly species exist. Second, there was a strong association between the fresh body mass of an odonate species and its encapsulation response and gregarine and water mite prevalences. Third, fresh body mass showed a very strong phylogenetic signal, whereas encapsulation and gregarine and water mite prevalences showed weak signals. Finally, when controlling for the effect of phylogeny, associations between the measured four traits lost their significance, indicating that phylogeny plays a role in these associations. It appears that the weight of different odonate species can be explained with the structure of phylogeny and weight has also a significant association with encapsulation response and gregarine and water mite prevalences. Encapsulation response has a weak phylogenetic signal, but it does not have a significant association with either type of parasitism. Both gregarine and water mite prevalences had a weak phylogenetic signal, and interestingly there was a positive correlation between gregarine and water mite prevalences. Even though encapsulation response showed only a weak phylogenetic signal, it may be strong enough to explain a certain degree of variation in gregarine and water mite prevalences. However, these results indicate that something else, perhaps a behavioural trait, links weight with encapsulation response and parasitism. Something that heavy odonates have or do, besides a stronger encapsulation response, lowers their parasitism rates.

As we predicted, we found considerable variation in the encapsulation response between different odonate species, but not between sexes. We also found that there is a weak phylogenetic signal in encapsulation response, indicating that phylogeny in itself can explain encapsulation levels to a certain degree and closely related species have similar levels of encapsulation response. Several previous studies have explored the encapsulation response of insects at the intra- or interspecies level, but with mixed results and with a wide range of explanations. Temperature has been indicated as a possible explanation of encapsulation differences [34], but it seems unlikely that it could explain species differences from the same geographical area. Food, or the lack of it, has explained variances in mosquitoes [21], and this might be a possible explanation also for our results. Larger odonates capture more prey on an absolute level compared with smaller species, so they may be able to use more resources for mounting an encapsulation response, but a larger body may also function as a larger energy reserve when faced with parasitism. Large size of a species may affect encapsulation response also through other means. In general, larger hosts are likely to be more attractive to parasites than smaller ones (e.g. [35]). If large hosts are more attractive than smaller ones to water mites especially, an early exposure as an odonate larvae (e.g. [36]) may induce a higher immune response as an adult host (immune priming theory, e.g. [3]). Unfortunately, no comparative studies have been done on larval odonates and their parasitism. However, larger odonate individuals have been found to have higher encapsulation responses [35] and fewer mites have been observed on large individuals [37], establishing the importance of host size in parasitism. In a study of two damselfly species [9], gender differences in encapsulation response did not match their equal parasitism rates, but were explained by differences in life histories. Ecological differences might also explain our results, because a wide range of differences, e.g. territoriality, geographical range area [38,39], population density, timing of emergence or the duration of larval stage, exist between our study species that are very likely to affect the immune system of a species. Especially in colder climates, the larval stage of damselflies is much shorter (1–2 years) compared with dragonflies (3 years or more) [25]. A study found that damselfly individuals had a stronger immune response as the season progressed [7]. In addition, it was found that an increase in resistance was accounted for by the daily temperatures in relation to emergence timing [40]. It is possible that this might have an effect on our results, because different odonate species were collected at different times during the summer and because on a general level smaller damselfly species have their flight times earlier in the season compared with larger dragonflies [41]. However, further studies are required to determine the extent of these factors. One likely candidate to explain encapsulation rates is parasitism, owing to the close relationship of immune defence and parasitism. It has been found in damselflies that the higher the parasitism rate, the higher the encapsulation response [6]. Opposite to this, species with low parasitism rates were found to have higher encapsulation rates [14,42]. To complicate things, it was recently found [13] that even though the parasitism levels of two odonate species were considerably different, the encapsulation response was similar. According to our data, there is a slight but not significant association between both gregarine and water mite parasitism and encapsulation response. Water mite parasitism showed a slightly stronger correlation, but that is expected because using an implant is designed to simulate the feeding tube of a larval water mite. It seems possible that a link exists between parasitism and encapsulation response. However, whether parasitism affects encapsulation response or vice versa, or whether a feedback loop of some kind exists, remains unclear.

As expected, there were significant differences in fresh body mass between species and sexes, females being heavier. Fresh body mass showed such a strong phylogenetical signal that weight differences between species could almost completely be explained through phylogeny. We also found that body mass associates very well with encapsulation response as well as gregarine and water mite parasitism. This could mean that a heavier species is able to mount a stronger encapsulation response, which in turn has an effect on parasitism. If we assume that encapsulating a foreign object requires the same amount of resources from the host independent of what species the host represents, the cost of encapsulation is less if the host can accumulate more resources compared with hosts with lower resource input. Larger odonates (dragonflies) are likely to acquire more resources compared with smaller ones (damselflies), and it is possible that they are also able to use more resources for encapsulation on an absolute level. This results in a higher encapsulation response and a better resistance against parasites. However, encapsulation response did not correlate significantly with gregarine or water mite parasitism, indicating that there is another underlying mechanism affecting parasitism rates. It is possible that the cuticle of large odonates is better at resisting water mites, or that the larger species eat larger prey and thus do not get infected with gregarines as often as smaller odonate species do.

Similar to our previous findings [22], we found significant differences between species in both gregarine and water mite parasitism. Interestingly, we found that males are less often infected with water mites than females, which is also similar to our previous findings [22], but this time the difference is significant. In addition, both gregarine and water mite prevalences showed weak phylogenetic signals, indicating that closely related species tend to have similar levels of gregarine and water mite parasites. There are other physiological factors besides body mass that might affect parasite levels in odonates. Hormones of the host, such as juvenile hormone, have been found to enhance the growth rate of water mites when applied to their dragonfly hosts [43]. This might indicate water mites' preference towards odonate species that have higher or specific levels of juvenile hormone. However, there are far too few studies done on this subject to properly speculate on the role of hormonal selection in host preference. In addition, we found a positive association between gregarine and water mite prevalences. This indicates that whenever a species harbours one parasite type, it is probable that it also has the other parasite type. It is possible that the existence of one parasite type in a host increases the host's susceptibility to other parasites. Unfortunately, there are only a few studies on the relationship between endo- and ectoparasites on a single host, so further studies are required to study this parasite association.

## 5. Conclusion

According to our data, weight of an odonate species can be explained by its phylogeny. This weight of an odonate species associates strongly with encapsulation response and gregarine and water mite parasitism, but why it does that is still unknown. Interestingly, encapsulation response does not associate with prevalence of either parasite, even though it is often considered a defence mechanism towards parasitism. In addition, the weak phylogenetic signals found on encapsulation response and gregarine and water mite parasitism indicate that closely related species may have a tendency to be similar in these three traits. To conclude, there seems to be something we have not studied yet, perhaps a behavioural trait or a physical attribute of an odonate species, which functions between the body weight of an odonate species and its parasitism.

## Supplementary Material

Appendix 1: Phylogeny of odonates This phylogenetic tree has the 22 odonate species used in this study. Branch length has been standardized to 1. We used four different published phylogenies to construct this and the references can be found from the main document.

## Supplementary Material

Appendix 2: R Script This file contains the R scripts we used in the phylogenetic analysis of this paper (Phylogenetic signal & PGLS models).

## Data accessibility

Data used in this study can be found in table 1. Raw data have been deposited in Dryad (http://dx.doi.org/10.5061/dryad.2v9g0) [44].
