# Supplementary figures and images for: Phylogeny affects host's weight, immune response and parasitism in damselflies and dragonflies
Source: R Soc Open Sci. 2016 Nov 9;3(11):160421. doi: 10.1098/rsos.160421 (PMC5180119; doi:10.1098/rsos.160421)

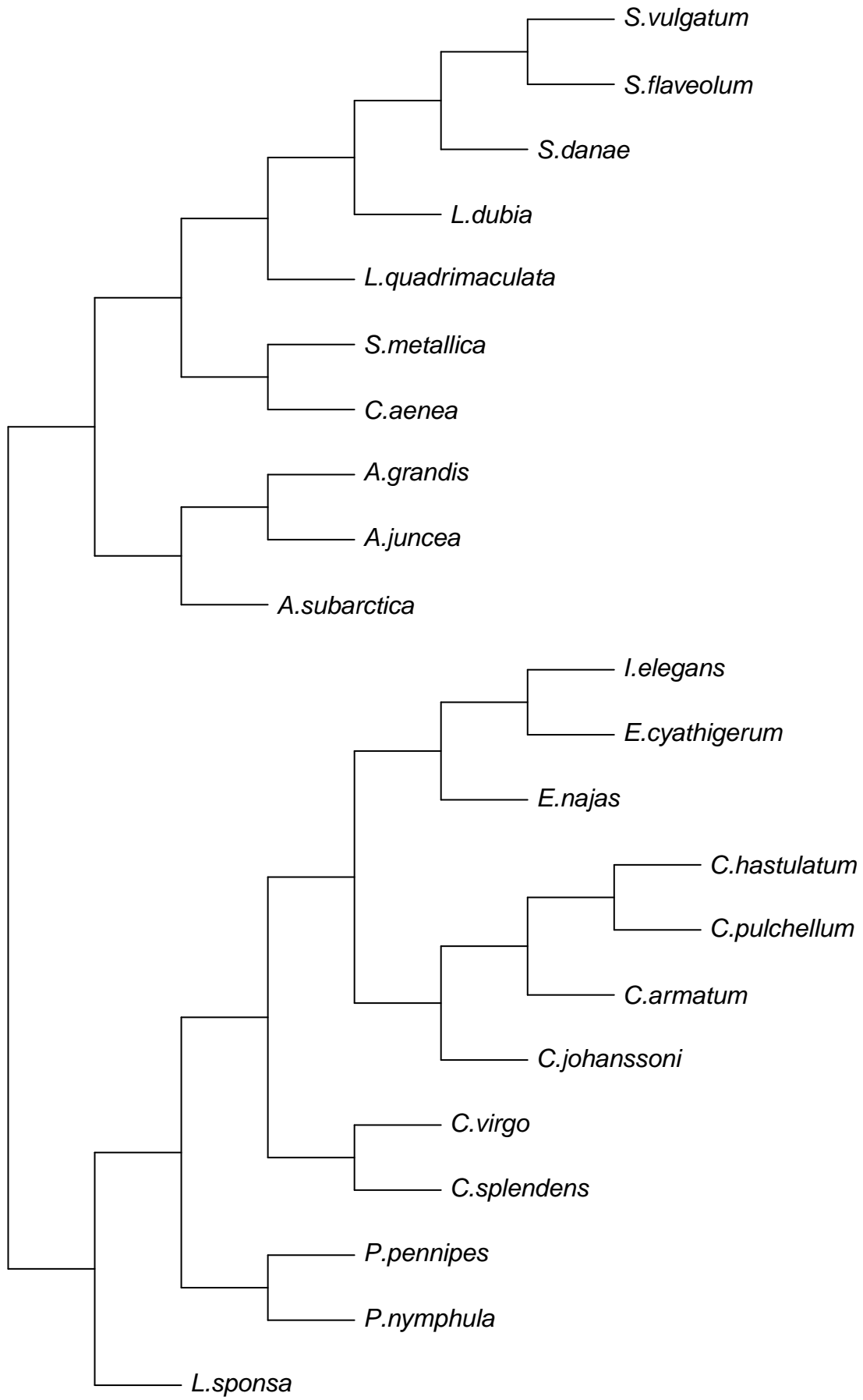

Supplement: Appendix 1: Phylogeny of odonates This phylogenetic tree has the 22 odonate species used in this study. Branch length has been standardized to 1. We used four different published phylogenies to construct this and the references can be found from the main document. [file rsos160421supp1.pdf]
