# Supplementary material for: Phylogeny affects host's weight, immune response and parasitism in damselflies and dragonflies
Source: R Soc Open Sci. 2016 Nov 9;3(11):160421. doi: 10.1098/rsos.160421 (PMC5180119; doi:10.1098/rsos.160421)
Supplement: Appendix 2: R Script This file contains the R scripts we used in the phylogenetic analysis of this paper (Phylogenetic signal & PGLS models). [file rsos160421supp2.docx]

#Here you can find the R script we used to find phylogenetic signals and calculate the PGLS #models.

#Finding a phylogenetic signal in the four traits we measured from damselflies and dragonflies #using Pagel's λ

#Set correct working directory with the tree file and datamatrix

setwd("F:/Data/phd/R ")

#Load packages

library(picante)

library(ape)

library(adephylo)

library(ade4)

library(phylobase)

library(geiger)

library(phytools)

#Load phylogenetic tree file:

tree <- read.tree("odonataphylogeny.txt")

#Load data

data <- read.csv("speciesdata.txt", header = TRUE, sep = ";", dec = ",")

#Phylogenetical signal of weight

weight<-data[,1]

names(weight)<-rownames(data)

phylosig(tree, weight, method="lambda", test=TRUE, nsim=999)

#Phylogenetical signal of encapsulation

encapsulation<-data[,2]

names(encapsulation)<-rownames(data)

phylosig(tree, encapsulation, method="lambda", test=TRUE, nsim=999)

#Phylogenetical signal of gregarines

gregarines<-data[,3]

names(gregarines)<-rownames(data)

phylosig(tree, gregarines, method="lambda", test=TRUE, nsim=999)

#Phylogenetical signal of water mites

watermites<-data[,4]

names(watermites)<-rownames(data)

phylosig(tree, watermites, method="lambda", test=TRUE, nsim=999)

#Phylogenetic generalized linear models of 22 different odonate species and their variables

#Set correct working directory with the tree file and datamatrix

setwd("F:/Data/phd/R")

#Load packages

library(ape)

library(phylolm)

library(geiger)

library(nlme)

library(caper)

#Load phylogenetic tree file:

tree <- read.tree("odonataphylogeny.txt")

#Load data

data <- read.csv("comparativespeciesdata.txt", header = TRUE, sep = ";", dec = ",")

#Comparative dataset

cdat <- comparative.data(data = data, phy = tree, names.col = "Species", vcv = TRUE)

#Creating different datasets

weight <- data$Weight

encapsulation <- data$Encapsulation

gregarines <- data$Gregarines

watermites <- data$Watermites

#PGLS models between two different variables

logweightencapsulation.pgls <- pgls(log(weight) ~ encapsulation, cdat)

logweightgregarines.pgls <- pgls(log(weight) ~ gregarines, cdat)

logweightwatermites.pgls <- pgls(log(weight) ~ watermites, cdat)

encapsulationgregarines.pgls <- pgls(encapsulation ~ gregarines, cdat)

encapsulationwatermites.pgls <-pgls(encapsulation~watermites, cdat)

gregarineswatermites.pgls <-pgls(gregarines~watermites, cdat)

#Summary of the different PGLS models

summary(logweightencapsulation.pgls)

summary(logweightgregarines.pgls)

summary(logweightwatermites.pgls)

summary(encapsulationgregarines.pgls)

summary(encapsulationwatermites.pgls)

summary(gregarineswatermites.pgls)
